# Reward Expectation Modulates Local Field Potentials, Spiking Activity and Spike-Field Coherence in the Primary Motor Cortex

**DOI:** 10.1523/ENEURO.0178-19.2019

**Published:** 2019-06-25

**Authors:** Junmo An, Taruna Yadav, John P. Hessburg, Joseph T. Francis

**Affiliations:** 1 Department of Biomedical Engineering, University of Houston, Houston, TX 77204; 2 Department of Physiology and Pharmacology, Robert F Furchgott Center for Neural and Behavioral Science, State University of New York Downstate Medical Center, Brooklyn, NY 11203

**Keywords:** α power, brain computer interface, mirror neurons, primary motor cortex, pulsed inhibition, reward

## Abstract

Reward modulation (M1) could be exploited in developing an autonomously updating brain-computer interface (BCI) based on a reinforcement learning (RL) architecture. For an autonomously updating RL-based BCI system, we would need a reward prediction error, or a state-value representation from the user’s neural activity, which the RL-BCI agent could use to update its BCI decoder. In order to understand the multifaceted effects of reward on M1 activity, we investigated how neural spiking, oscillatory activities and their functional interactions are modulated by conditioned stimuli related reward expectation. To do so, local field potentials (LFPs) and single/multi-unit activities were recorded simultaneously and bilaterally from M1 cortices while four non-human primates (NHPs) performed cued center-out reaching or grip force tasks either manually using their right arm/hand or observed passively. We found that reward expectation influenced the strength of α (8–14 Hz) power, α-γ comodulation, α spike-field coherence (SFC), and firing rates (FRs) in general in M1. Furthermore, we found that an increase in α-band power was correlated with a decrease in neural spiking activity, that FRs were highest at the trough of the α-band cycle and lowest at the peak of its cycle. These findings imply that α oscillations modulated by reward expectation have an influence on spike FR and spike timing during both reaching and grasping tasks in M1. These LFP, spike, and spike-field interactions could be used to follow the M1 neural state in order to enhance BCI decoding (An et al., 2018; Zhao et al., 2018).

## Significance Statement

Knowing the subjective value of performed or observed actions is valuable feedback that could be used to improve the performance of an autonomously updating brain-computer interface (BCI). Reward-related information in the primary motor cortex (M1) may be crucial for more stable and robust BCI decoding (Zhao et al., 2018). Here, we present how expectation of reward during motor tasks, or simple observation, is represented by increased spike firing rates (FRs) in conjunction with decreased α (8–14 Hz) oscillatory power, α-γ comodulation, and α spike-field coherence (SFC), as compared to nonrewarding trials. Moreover, a phasic relation between α oscillations and FRs was observed where FRs were found to be lowest and highest at the peak and trough of α oscillations, respectively.

## Introduction

The brain is a highly adaptable learning machine, and at least partially learns via reinforcement learning (RL) mechanisms. As a main function of the brain is to move the individual through their environment in a manner that maximizes reward and minimizes punishment, we hypothesized that one would see neural dynamics reminiscent of various aspects of a RL machine (Tarigoppula et al., 2018), and we expected to see this at every level of the neural representation from mesoscopic/macroscopic EEG (i.e., electroencephalography) down to single units (Marsh et al., 2015; McNiel et al., 2016; An et al., 2018), and conducted this work to explore this hypothesis. Another motivation for our work is that we hope to use reward related information from a user’s brain, such as state value information, or reward prediction error, toward autonomously updating brain-computer interface (BCI) agents that use RL update rules. Thus, if we can better understand the neural signatures of reward expectation, we can better move toward such autonomously updating BCIs.

Recently, cross-frequency coupling (CFC) has been used to measure statistical correlations between different frequency bands of macroscopic (slow-frequency oscillations) and/or mesoscopic (high-frequency oscillations) local field potential (LFP) oscillations (Soltesz and Deschênes, 1993; Bragin et al., 1995). CFC has been considered an important measure of cognitive information processing in different brain regions (Canolty and Knight, 2010). Phase-to-phase coupling (Palva et al., 2005; Belluscio et al., 2012), phase-to-amplitude coupling (PAC; Mormann et al., 2005; Canolty et al., 2006; Tort et al., 2008; Colgin et al., 2009), and amplitude-to-amplitude coupling (Bruns et al., 2000) are different methods in which CFC can be analyzed (Jensen and Colgin, 2007; Canolty and Knight, 2010). In particular, PAC between the amplitude of high-frequency oscillations and the phase of low-frequency oscillations has been observed in cognitive tasks in both human (Canolty et al., 2006; Axmacher et al., 2010) and animal models (Buzsáki et al., 2003; Lakatos et al., 2005; Tort et al., 2008). In addition, the correlation between microscopic (spike trains) and macroscopic and/or mesoscopic (LFPs) scales has been considered to play an important functional role in neural processing (Fries et al., 2001; Jarvis and Mitra, 2001). Several studies have employed spike-field coherence (SFC), the phase dependency between spikes and LFPs, to investigate the communication and synchronization within neuronal groups of the same or different cortical regions (Womelsdorf et al., 2006; Witham et al., 2007; Fries et al., 2008; Pesaran et al., 2008; Gregoriou et al., 2009; Jutras et al., 2009; Chalk et al., 2010).

For many years, α (8–14 Hz) oscillations were thought to serve a non-functional purpose, where the power in this band reflected the opened or closed state of the eyes (Adrian and Matthews, 1934). However, several recent studies have shown the functional role of α oscillations and their importance in cognitive processing. Specifically, α oscillations reflect inhibitory activity in visual and auditory attention (Foxe et al., 1998; Thut et al., 2006; Rihs et al., 2009; Kerlin et al., 2010), perception (VanRullen and Koch, 2003), and working memory (Jensen et al., 2002; Sauseng et al., 2005; Jensen and Mazaheri, 2010) tasks. However, it is less clear how reward expectation affects these oscillations in the primary motor cortex (M1).

To better understand reward-related effects on M1, we conducted the present study where we simultaneously recorded neural spiking activity (single- and multi-units) and LFPs from contra/ipsilateral M1 in non-human primates (NHPs) while they performed cued trial value center-out reaching tasks and grip force tasks. We have previously shown that reward modulates single unit activity, population firing rates (FRs), and LFP power in M1 while NHPs either performed or observed a single target center-out reaching task (Marsh et al., 2015), work that has since been corroborated and extended by others (Ramkumar et al., 2016; Ramakrishnan et al., 2017). To further understand the impact of reward expectation on other aspects of M1 neural activity, we studied power spectral density (PSD), phase-to-amplitude comodulation, SFC, and phase correlation with spiking activity.

## Materials and Methods

### Surgery

A rhesus macaque [NHP P (female): *Macaca mulatta*] and three bonnet macaques [NHPs A (male), S (male), and Z (female): *Macaca radiata*] were implanted with 96-channel microelectrode Utah arrays (10 × 10 array consisting of 1.5 mm in length electrodes spaced 400 μm, Blackrock Microsystems, LLC.) in the M1 region associated with their right hand and forearm. NHPs A, S, and P were implanted in the contralateral M1 with respect to the right arm, which all NHPs used to perform the manual tasks. NHP Z was previously implanted twice in the contralateral M1, therefore the array was implanted in ipsilateral M1 for this study. All studies and procedures were approved by the Institutional Animal Care and Use Committee at the State University of New York (SUNY) Downstate Medical Center and complied with the National Institutes of Health Guide for the Care and Use of Laboratory Animals guidelines. The surgical procedures used in the experiment were the same as those as described in our previous work (Chhatbar et al., 2010; Marsh et al., 2015). In brief, veterinary staffs from the SUNY Downstate Division of Comparative Medicine performed general anesthesia and animal preparation. Aseptic conditions were maintained during the course of surgery. Anesthesia was induced with ketamine and maintained using isoflurane and fentanyl. To prevent inflammation, dexamethasone was used during the surgical procedure.

The first surgery for each animal was the implantation of a back post, a titanium post (Crist Instrument Co., Inc.) implanted onto the caudal aspect of the NHP’s cranium to attach to the primate chair during training and recording. The post was placed on the caudal aspect of the skull, holes drilled and tapped (Synthes 2.0 mm drill bit, Synthes 2.0 mm tap) and affixed with 6- or 8-mm screws (Synthes, 2.7 mm in diameter, titanium) depending on the thickness of the NHP’s skull.

For microelectrode implantation, the animal was prepared for surgery in the same way. A rostrally placed front post (titanium, Crist Instrument Co., Inc.) was affixed similarly to the back post, to serve as a platform for the electrode connectors. An ∼2 × 3-cm craniotomy window was then created using a dremel tool with conical tip (Dremel Multipro), over the cortical areas of interest. The dura mater was reflected, and the target locations were identified visually with cortical landmarks. To confirm the location of somatosensory cortex (S1), an electrode (Michigan probe, 4-shank 32 channel silicone array, NeuroNexus Technologies, Inc.) was lowered stereotactically into the cortex. A lab member then stimulated the animal by tapping the contralateral hand and arm, with the electrode output represented audibly through loudspeakers routed through a TDT recording system (Tucker Davis Technologies, Inc.). A 96-channel microelectrode array (Blackrock Utah array, platinum/iridium, 1.5 mm in length) was placed in the determined S1 location, and a pneumatic piston (pneumatic control box, Cyberkinetics Neurotechnology Systems, Inc.) used to secure the array into place in the cortex. The M1 implanted location was immediately across the central sulcus, and put into place in the same manner.

Electrode wires were gathered together and routed to one corner of the craniotomy, and then along the front post to the electrode connectors, which were placed into a plastic frame attached to the front post. Once all the connectors were affixed the dura was sutured back into place, and the bone fragment from the craniotomy window placed above. The bone was affixed with a titanium mesh and bone screws (1.9 mm in diameter, 4 mm in length titanium self-tapping screws, Bioplate Inc.) on the skull and on the bone fragment. Dental acrylic (Palacos, Zimmer Biomet) was used to attach the electrode wires to the skull, and additionally to create a protective layer between the craniotomy and the front post; so, the NHP could not interfere with the wires. NHPs were given a six-week rest period following back post implantation to allow for osseointegration before resuming training and recording, and two weeks following microelectrode implantation to allow for the site to heal and cortical inflammation to reduce.

### Cued center-out reaching task

**Figure 1.**
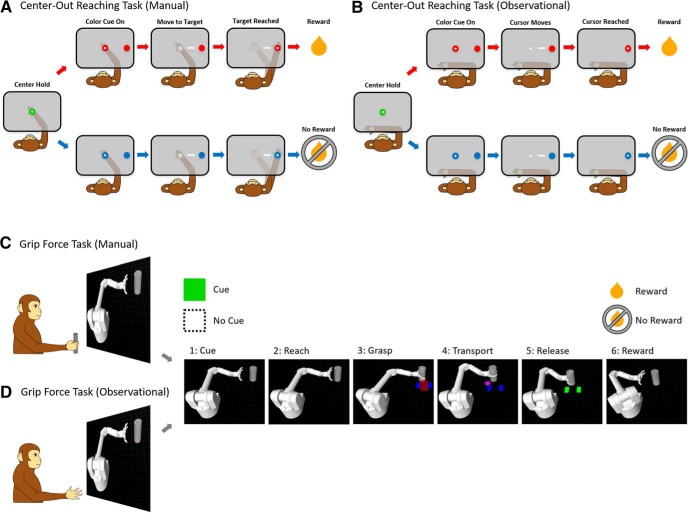
Center-out reaching tasks and grip force tasks. Schematic of (***A***) manual and (***B***) observational task during the cued center-out reaching tasks as well as for cued manual (***C***) and observational (***D***) grip force tasks.

Two NHPs (A and Z) were trained to perform a center-out delayed hold reaching task with their right arm using a two-link robotic exoskeleton (KINARM, BKIN Technologies Ltd.) for the manual task, as shown in Figure 1*A*. They were also trained to observe a feedback cursor moving automatically with a constant speed toward the target without performing physical effort during the observational task, as shown in Figure 1*B*. During the manual task, the NHPs would have to hold their hand on a central target for 325 ms, following a color cue period of 100–300 ms dependent on the NHPs temperament. During the color cue period the peripheral targets color and the hold target’s color indicated the trials value that is rewarding or nonrewarding. The NHP had to wait another 325–400 ms until the go cue, which was the disappearance of the hold target. For a successful trial, the NHP had to reach and hold on the peripheral target for 325 ms. Every successful reach in rewarding trials resulted in a juice reward to the NHP, whereas reward was withheld on nonrewarding successful trials. If a nonrewarding trial was unsuccessful, it was repeated to encourage NHPs to make successful movements. During observational tasks, NHPs observed passively while a feedback cursor moved automatically from center to peripheral target at a constant speed (∼1 cm/s). Visual color cues were similar to the manual task, and informed the NHP about the rewarding or nonrewarding trial value if successful. During manual and observational tasks, rewarding and nonrewarding trials were presented in a random order. There was one exception for NHP A during observational tasks where trials followed a set structure or rewarding followed by nonrewarding and repeating.

### Cued grip force task

Two NHPs (S and P) were trained to perform cued grip force tasks sitting comfortably in a primate-training chair (BKIN Technologies Ltd.) either manually (Fig. 1*C*) or observationally (Figure 1*D*). The task consisted of a virtual robotic arm, modeled as a Barrett WAM Arm and Hand (Barrett Technology) in Gazebo on ROS (Robot Operating System) that reached toward a virtual cylindrical object. When the robotic arm reached the object, the NHP was required to apply and maintain a visually indicated amount of force on a custom-made manual gripper (force transducer) which was composed of a metal bar, a cylindrical plastic frame, and a force transducer (FC2231-0000-0100-L, Measurement Specialties, Inc.) to measure grip force while the cylinder was transported to the target position by the WAM simulation. Once at the target position, the NHP would release the manual gripper, and the robotic hand would release the object, and the arm moved back to the neutral starting position. The target force was indicated with a pair of blue rectangles in the virtual environment, where the width of the rectangles represented the upper and lower bounds of the required grip force. The actual magnitude of force applied by the NHP was shown as a red rectangle that expanded as the force increased. The correct amount of grasping force was maintained by keeping the red rectangle within the upper and lower bounds of the blue rectangle. For a trial to be considered successful two conditions had to be met. The first was the application of appropriate force during the transport of the object to the target location, and the second condition was the release at the end of object’s transfer. Based on whether a trial was cued rewarding or nonrewarding, the NHP, respectively, received or did not receive a juice reward at the end of a successful trial. The visual color cue was shown at the beginning of each trial and remained visible throughout the trial. This trial progression was divided into six timeframes for analysis: (1) cue, when the cue was presented; (2) reach, when the virtual arm approached the target; (3) grasp, when the NHP applied force to the manual gripper so the virtual hand grasped the object; (4) transport, where the object automatically moved to the target location while the NHP maintained grip force; (5) release, when the gripper was released and the virtual hand released the object; and (6) reward or non-reward, when the NHP received a juice reward for successful completion of a rewarding trial, did not receive reward for completion of a nonrewarding trial, or did not receive reward for the unsuccessful completion of either trial type.

The structure of rewarding and nonrewarding trials was completely predictable in some recording blocks, where trials alternated between the two. The remainder of the blocks was partially predictable, where 50%, 75%, or 90% of the trials were rewarding, and the trial type was selected pseudorandomly with this bias. In the observational task, the mechanics of the task were the same, but the NHP had to passively observe the robot performing an automatic grasp and transport of the cylinder instead of manually applying force.

### Neural recording

Spike trains and LFPs were recorded simultaneously from M1 cortices using a Multichannel Acquisition Processor recording system (MAP, Plexon Inc.) while the NHPs performed center-out reaching tasks and grip force tasks. The recorded neural signals were bandpass-filtered from 170 Hz to 8 kHz for spike trains and from 0.7 to 300 Hz for LFPs using a linear finite impulse response (FIR) filter, and sampled at 40 kHz for spikes and 2 kHz for LFPs using a MAP system. Offline spike sorting was performed using a custom-made sorting template in the commercial software, Offline Sorter (Plexon Inc.). We analyzed single/multi-unit activity and LFPs recorded from the contralateral M1 area of NHPs A, S, and P as well as the ipsilateral M1 area of NHP Z.

### Analysis of PSD

To probe whether reward expectation modulates the power of neural oscillations, PSD of the LFPs was estimated using the Welch periodogram method with 75% overlapping Hamming windows (Welch, 1967). LFPs recorded from 32 channels were preprocessed by first removing the line noise (60 Hz) from every LFP channel using a second-order Butterworth notch filter, following which each channel was z-scored. Then, LFP channels with signal-to-noise ratio (SNR) below 5 were excluded from further analysis. SNR was calculated as the ratio of peak-to-peak amplitude (*A_Pk-Pk_*) of the averaged LFP (*LFP_Avg_*) and twice the SD of the residual signal (given by the difference of LFP on *i^th^* channel and *LFP_Avg_*) as in Equation 1, where *i ∊ [1,32]* (Koralek et al., 2013)

(1) $$SNR_{i}=\frac{A_{pk-pk}}{2*SD(LFP_{i}-LFP_{Avg})}$$

This SNR calculation was performed separately for each NHP dataset. Based on SNR criterion, the number (average ± SD) of LFP channels used for manual task datasets was 27.5 ± 1.0, 26.0 ± 2.7, 30.7 ± 0.6, and 24.0 ± 0.0 in NHPs A, Z, S, and P, respectively. For observational task datasets, 24.0 ± 2.0, 24.3 ± 3.5, 30.0 ± 0.0, and 27.0 ± 5.8 of LFP channels were used for NHPs A, Z, S, and P, respectively. Selected LFP channels were averaged and used for PSD estimation. For comparison between rewarding and nonrewarding trials, each PSD was then normalized by dividing the power at each frequency by the average of all power from 0.5 to 100 Hz (Colgin et al., 2009). The trial-averaged PSD in the α-band (8–14 Hz) was employed to compare rewarding to nonrewarding trials while NHPs performed all tasks.

### Analysis of CFC

To quantify the CFC in LFPs and test its relationship with reward expectation, the PAC method [Kullback–Leibler-based modulation index (MI)] was used as proposed by Tort et al. (2008). PAC analysis can be described briefly in the following manner (Tort et al., 2008). PAC appears when the amplitude of fast frequency oscillations is modulated by the phase of slow-frequency oscillations (Lakatos et al., 2005; Canolty et al., 2006; Jensen and Colgin, 2007; Tort et al., 2008). First, the averaged LFP was bandpass-filtered at a low-frequency band (8 Hz to 20 Hz) for phase and a high-frequency band (25–100 Hz) for amplitude. Second, the phase of the low-frequency band and amplitude of the high-frequency band were extracted from the above filtered LFP by applying Hilbert transform. Third, the strength of amplitude comodulation by phase was computed as the MI measure at each phase frequency and amplitude frequency pair. The MI was calculated in steps of 0.5 Hz for phase frequency and 5 Hz for amplitude frequency using the normalized entropy measure as shown in Equations 2–4 (for review, see Tort et al., 2008).

(2) $$p_{j}=\frac{<A_{f_{A}}>\phi_{f_{p}}(j)}{\overset{N}{\underset{j=1}{\sum }}<A_{f_{A}}>\phi_{f_{p}}(j)}$$

(3) $$H=-\overset{N}{\underset{j=1}{\sum }}p_{j}\;\log\left(p_{j}\right)$$

(4) $$MI=\frac{H_{\max}-H}{H_{\max}}$$

Here, *N* is the total number of phase bins (*N* = 18; each bin has 20° interval ranging from 0° to 360°). < *A_f_A__* > Ø*_f_p__*(*j*) indicates the mean of the instantaneous amplitude *A_f_A__* value over the phase bin *j* of phase frequency Ø*_f_p__*(*j*). *H_max_* denotes the maximum possible entropy value (*H_max_* = log*N*).

Using this PAC method, we computed the strength of phase-to-amplitude comodulation in rewarding and nonrewarding trials for center-out reaching tasks and grip force tasks and compared them for statistically significant differences.

### Analysis of SFC

To examine whether reward expectation modulates the coherence between spike trains and LFPs, we conducted SFC analysis. SFC is commonly used to compute phase synchronization between point process data (e.g., spike trains) and continuous data (e.g., LFPs). To rule out the possibility that spikes might have contaminated LFPs (Waldert et al., 2013), all LFPs from selected channels (higher SNR channels) were averaged, then the power spectra of the binned point processes (single unit activity, binned at 1 ms) and channel-averaged LFP were computed using the multitaper estimation method. For this purpose, we used a Chronux built-in function, *coherencycpb()*, with nine tapers and a time-bandwidth (TW) value equal to 5. The coherence between spikes and LFPs was computed using the formula as shown in Equation 5 (Fries et al., 2001; Jarvis and Mitra, 2001)

(5) $$C_{SL}=abs(\frac{{\text{S}}_{SL}}{\sqrt{S_{S}*S_{L}}})\;$$

Here, *S_SL_* indicates the cross-spectrum between spikes and LFPs, and *S_S_* and *S_L_* indicate the autospectra of spikes and LFPs, respectively. An SFC value (*C_SL_*) of zero indicates the absence of phase synchronization between spikes and LFPs, and one indicates the perfect synchronization between them. The trial-averaged SFC was analyzed over the frequency band (0.5–100 Hz) and the trial-averaged SFC at the α frequency band was used to compare rewarding trials to nonrewarding trials while NHPs performed the aforementioned tasks.

### Relation between α cycle and neural spiking

The interaction between the α-band cycle and neural spiking activity was measured as follows (Haegens et al., 2011). First, we bandpass-filtered LFP oscillations from 8 to 14 Hz. Second, the phase of the α-band LFP was obtained by applying the Hilbert transform. Third, we divided the α-band cycle into six equally sized phase bins of 60° each. Fourth, each unit’s FR was normalized across the six phase bins using the min-max scaling method, obtained by *Normalized FR = [FR – min(FR)]/[max(FR) – min(FR)]*; last, the average FR in each phase bin was computed over a population of units and represented with respect to the corresponding phase of α oscillations.

### Statistical analysis

All statistical analyses were performed using MATLAB R2017b (MathWorks Inc.). The nonparametric two-sided Wilcoxon signed-rank (paired) test [significance level (*p*) = 0.05] was used to evaluate significant differences of PSD, PAC, SFC, and spike FRs between rewarding and nonrewarding trials for all tasks. To test the statistical significance of changes in FR with respect to the α-band phase bins, we performed one-way ANOVA with Bonferroni *post hoc* multiple comparison (significance level: α = 0.05) for rewarding and nonrewarding trials individually. The *F*-statistic value (from one-way ANOVA) and *p* value computed between the phase bins of low and high FRs (from two-sample *t* test) were observed for different tasks and trial types. Our analysis described in this paper focused on the α-band as this was the only band to show significant differences for all NHPs and all tasks using the two-sided Wilcoxon signed-rank test.

## Results

To investigate how cued reward expectation influences neural activity in M1 cortex, we performed PSD and PAC analyses with LFPs, computed spike FR, and quantified SFC on neural spikes and LFP data obtained from contralateral (NHPs A, S, and P) and ipsilateral (NHP Z) M1 across center-out reaching tasks and grip force tasks. In addition, we investigated how α-band oscillations correlate with spike FR. As mentioned in the methods, task structure could either be completely predictable or random with a bias. For NHP A’s observational task, rewarding and nonrewarding trials were presented as a sequence alternating between the two while all the other tasks for all NHPs were randomly ordered with a bias which varied based on the percentage of reward bias.

### Reward expectation modulates α power

**Figure 2.**
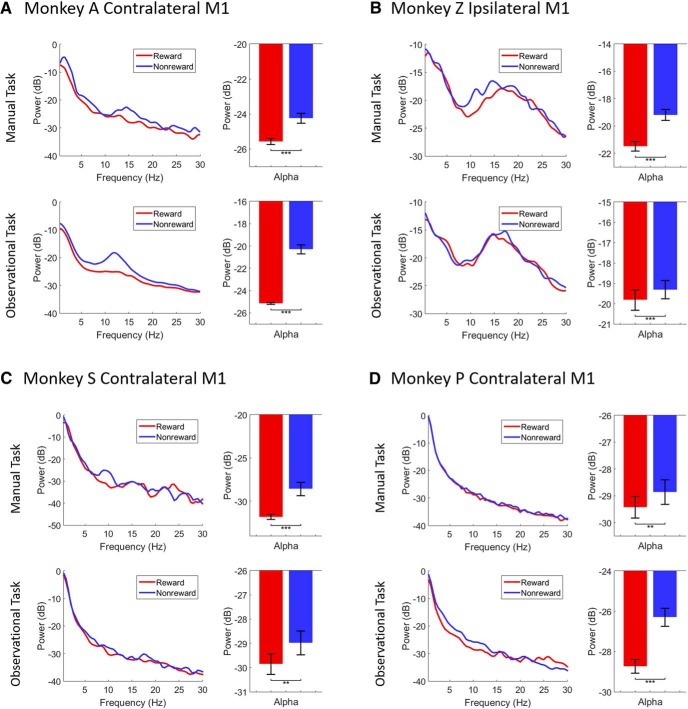
PSD plots (left column in each subplot) of LFPs for rewarding (red) and nonrewarding (blue) trials across manual (upper row in each subplot) and observational (lower row in each subplot) tasks during center-out reaching (***A***, ***B***) tasks and grip force (**C**, ***D***) tasks. Bar graphs show significant differences of α (8-14 Hz, right column in each subplot) band (***p < 0.001, **p < 0.01; Wilcoxon signed-rank test). Error bars in the bar plots represent SEM.

For PSD analysis, we used a postcue-onset period of 800 ms for all tasks. Figure 2 displays the normalized PSD plots (left column in each subplot) and bar plots for the α-band (right column in each subplot). Shown are significant differences of α (8–14 Hz binned at 0.5 Hz) LFPs for rewarding (red) and nonrewarding (blue) trials across manual (upper row in each subplot) and observational (lower row in each subplot) tasks during both center-out reaching tasks (NHPs A and Z as shown in Fig. 2*A*,*B*) and grip force tasks (NHPs S and P as shown in Fig. 2*C*,*D*).

During the manual reaching task from NHP A’s contralateral M1 (Fig. 2*A*, upper row), the PSD in the α-band was significantly higher during nonrewarding trials than rewarding trials (Wilcoxon signed-rank test, *p* < 0.001, *z-*statistic = 3.11, *n* = 13). Furthermore, for the observational task (Fig. 2*A*, lower row), we found similar patterns of neural activation. Similar to the manual task, α-band PSD was significantly higher during nonrewarding than rewarding trials for the observational task (Wilcoxon signed-rank test, *p* < 0.001, *z-*statistic = 3.18, *n* = 13). During the manual-reaching task from NHP Z’s ipsilateral M1 (Fig. 2*B*, upper row), again the α power was significantly higher during nonrewarding than rewarding trials (Wilcoxon signed-rank test, *p* < 0.001, *z-*statistic = 3.18, *n* = 13). During the observational task (Fig. 2*B*, lower row), α PSD showed a significant increase during nonrewarding trials as well (Wilcoxon signed-rank test, *p* < 0.001, *z-*statistic = 3.18, *n* = 13).

Data from the manual grip force task from NHP S’s contralateral M1 (Fig. 2*C*, upper row) showed that α PSD was significantly higher for nonrewarding than rewarding trials (Wilcoxon signed-rank test, *p* < 0.001, *z-*statistic = 3.11, *n* = 13). Similarly, for the observational task (Fig. 2*C*, lower row), α PSD was significantly higher for nonrewarding than rewarding trials (Wilcoxon signed-rank test, *p* < 0.01, *z-*statistic = 2.76, *n* = 13). During both manual (Fig. 2*D*, upper row) and observational (Fig. 2*D*, lower row) grip force tasks from NHP P’s contralateral M1, α PSD was significantly higher for nonrewarding than rewarding trials (Wilcoxon signed-rank test, *p* < 0.01, *z-*statistic = 2.55, *n* = 13 for manual task and *p* < 0.001, *z-*statistic = 3.18, *n* = 13 for observational task). The overall PSD results in Figure 2 indicate that the α power of LFP oscillations were modulated by cued reward expectation. It also indicates that α power was significantly increased for nonrewarding trials as compared to rewarding trials for both manual and observational variations of arm reaching tasks and hand grasping tasks.

### Reward expectation modulates α-γ comodulation

**Figure 3.**
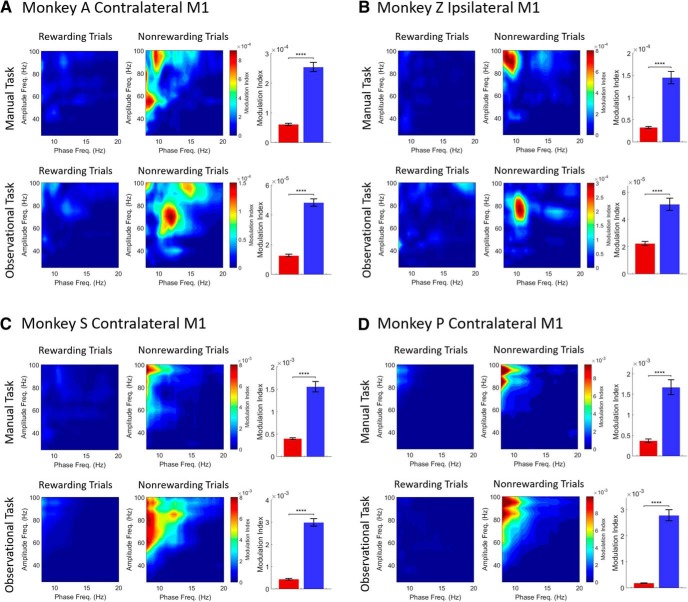
Comodulograms showing the MI for rewarding (left column in each subplot) and nonrewarding trials (middle column in each subplot) across manual (upper row in each subplot) and observational tasks (lower row in each subplot) for contralateral (***A***, ***C***, ***D***) and ipsilateral (***B***) M1 cortices. Bar graphs (right column in each subplot) show significant differences of α-γ MI values for rewarding (red) and nonrewarding (blue) trials (****p < 0.0001; Wilcoxon signed-rank test). Error bars in the bar plots represent SEM. ***A***, ***B*** are for the center-out reaching tasks, and ***C***, ***D*** are for the grip force tasks.

To determine whether the phase of low-frequency oscillations was related to the amplitude of high-frequency oscillations in M1 during our reward cued experiments (Fig. 1), we computed phase-to-amplitude comodulation during a postcue-onset period of 800 ms both for rewarding and nonrewarding trials across all tasks. Figure 3 displays phase-to-amplitude comodulogram plots for rewarding trials (left column in each subplot) and nonrewarding trials (middle column in each subplot) across manual (upper row in each subplot) and observational (lower row in each subplot) tasks for contralateral (Fig. 3*A*,*C*,*D*) and ipsilateral (Fig. 3*B*) M1 cortices. In addition, the bar plot distributions (right column in each subplot) at phase frequencies of 8–14 Hz (binned at 0.5 Hz) and at amplitude frequencies of 30–100 Hz (binned at 5 Hz) in comodulograms showed significant differences of α-γ MI values for rewarding (red) and nonrewarding (blue) trials. During the manual reaching task from NHP A’s contralateral M1 (Fig. 3*A*, upper row), α-γ PAC was significantly greater for nonrewarding trials than rewarding trials (Wilcoxon signed-rank test, *p* < 0.0001, *z-*statistic = 11.12, *n* = 195) when the phase is in the α frequency at ∼10 Hz. For the observational task (Fig. 3*A*, lower row), α-γ PAC was also significantly higher during nonrewarding trials than during rewarding trials (Wilcoxon signed-rank test, *p* < 0.0001, *z-*statistic = 11.30, *n* = 195) at α phase within the frequency band of 10–14 Hz. Similar trends were observed for NHP Z’s ipsilateral M1 (Fig. 3*B*), where α-γ comodulation at phase frequencies of 8–10 Hz for manual task and at phase frequency of ∼10 Hz for observational task during nonrewarding trials were significantly greater than rewarding trials (Wilcoxon signed-rank test, *p* < 0.0001, *z-*statistic = 10.03, *n* = 195 for manual task and *p* < 0.0001, *z-*statistic = 5.84, *n* = 195 for observational task).

The results for the center-out reaching tasks indicate that the strength of α-γ frequency PAC analyzed with the averaged LFP oscillations recorded from contralateral and ipsilateral M1 were modulated by reward expectation in both the presence (for manual) and absence (for observational) of arm reaching movements.

Qualitatively similar PAC results were observed in the grip force tasks. In Figure 3*C*, α-γ comodulations for both the manual (upper row) and observational (lower row) grip force tasks from NHP S’s contralateral M1 were significantly higher during nonrewarding trials than rewarding trials (Wilcoxon signed-rank test, *p* < 0.0001, *z-*statistic = 12.08, *n* = 195 for manual task and *p* < 0.0001, *z-*statistic = 12.11, *n* = 195 for observational task). In Figure 3*D*, the PAC results of NHP P also showed significantly higher α-γ comodulation for nonrewarding trials than rewarding trials for both the manual (upper row) and observational (lower row) tasks (Wilcoxon signed-rank test, *p* < 0.0001, *z-*statistic = 12.11, *n* = 195 for manual task and *p* < 0.0001, *z-*statistic = 12.11, *n* = 195 for observational task). Similar to PAC results for the center-out reaching tasks, our results demonstrate that the strength of α-γ comodulation was influenced by reward expectation during both manual and observational grip force tasks.

### Reward expectation modulates SFC

**Figure 4.**
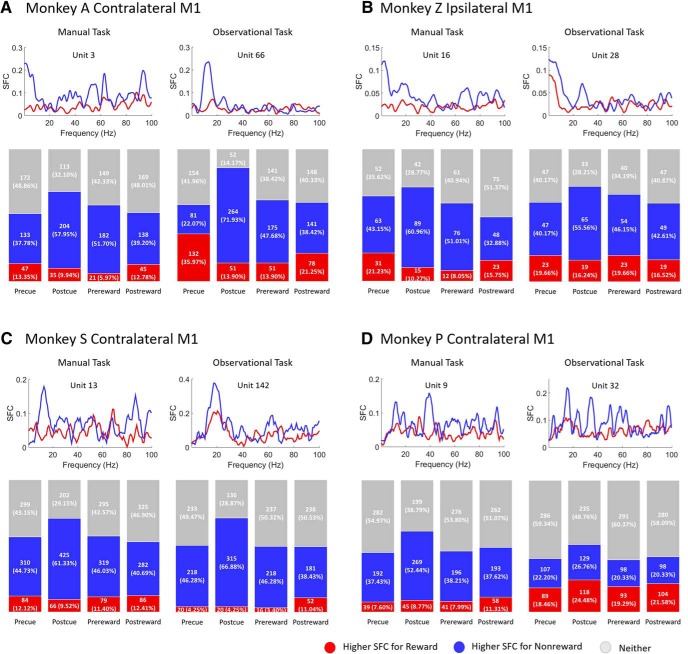
SFC plots (upper row in each subplot) for sample units for rewarding (red) and nonrewarding (blue) trials across manual (left column in each subplot) and observational (right column in each subplot) tasks for contralateral (***A***, ***C***, ***D***) and ipsilateral (***B***) M1 cortices. Bar charts (lower row in each subplot) represent the population of significantly different M1 units for SFC values in α-band (8–14 Hz) during rewarding (red) and nonrewarding (blue) trials, and those with no significant difference (gray; Wilcoxon signed-rank test, *p* < 0.05). Each column in the bar chart represents precue (before cue, 500 ms), postcue (after cue, 800 ms), prereward (before reward, 500 ms), and postreward (after reward, 500 ms) periods. ***A***, ***B*** are for the center-out reaching tasks, and ***C***, ***D*** are for the grip force tasks.

To investigate whether α oscillations are related to phase synchronization between spikes and LFPs, reward-related changes in the α-band (8–14 Hz) SFC were estimated during the center-out reaching tasks and grip force tasks. Figure 4 shows SFC plots (upper row in each subplot) for sample units (with specific unit number) during a postcue (after cue) period of 800 ms for rewarding (red) and nonrewarding (blue) trials for contralateral (NHPs A, S, and P) and ipsilateral (NHP Z) M1 cortices across all tasks. Additionally, Figure 4 displays the population (lower row in each subplot) of significantly different units for SFC values in the α-band during rewarding (red) and nonrewarding (blue) trials, or neither (gray; Wilcoxon signed-rank test, *p* < 0.05). From left to right in the bar charts (lower row in each subplot), each column represents the precue (before cue, 500 ms), postcue (after cue, 800 ms), prereward (before reward, 500 ms), and postreward (after reward, 500 ms) time windows. In NHP A’s contralateral M1 during manual center-out reaching tasks (Fig. 4*A*, left column), 37.8% (133 of 352 for precue), 58.0% (204 of 352 for postcue), 51.7% (182 of 352 for prereward), and 39.2% (138 of 352 for postreward) of M1 units had significantly higher trial-averaged SFC during nonrewarding trials than rewarding trials. For observational center-out reaching tasks (Fig. 4*A*, right column), the percentages are 22.0% (81 of 367 for precue), 71.9% (264 of 367 for postcue), 47.7% (175 of 367 for prereward), and 38.4% (141 of 367 for postreward) of M1 units. Similar results were seen in NHP Z’s ipsilateral M1 during the center-out reaching tasks. The detailed breakdown of the results can be seen in Figure 4*B*, left column for manual task and right column for observational task.

As with NHPs A and Z, NHP S also showed significantly higher SFC percentages in nonrewarding trials for both manual and observational grip force tasks shown in Figure 4*C*. This pattern remains consistent in NHP P for manual tasks (Fig. 4*D*, left column), but with less significance for observational tasks (Fig. 4*D*, right column). Furthermore, the postreward (after reward delivery) period in observational tasks for NHP P showed a slightly higher percentage of SFC for rewarding than nonrewarding trials. Overall, the results in Figure 4 indicate that M1 units are significantly modulated by reward expectation for α-band SFC during all reaching tasks and grasping tasks.

### Reward expectation modulates spike FR

**Figure 5.**
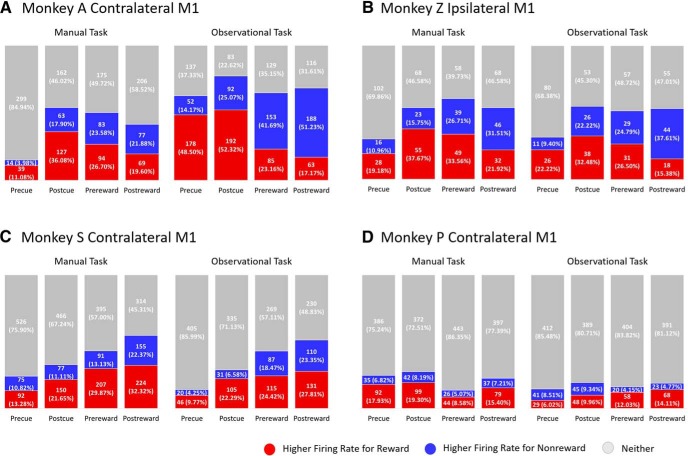
Total population of M1 units that had significantly higher FRs for rewarding (red), nonrewarding (blue) trials, or neither (gray) across manual (left column) and observational (right column) tasks for contralateral (***A***, ***C***, ***D***) and ipsilateral (***B***) M1 cortices (Wilcoxon signed-rank test, *p* < 0.05). Each column in the bar chart represents precue (500 ms), postcue (800 ms), prereward (500 ms), and postreward (500 ms) periods. ***A***, ***B*** are for the center-out reaching tasks, and ***C***, ***D*** are for the grip force tasks.

To examine the reward expectation modulation of neural spiking in M1, we computed the FR of M1 units during the center-out reaching tasks and grip force tasks. For each M1 unit, FR was computed using 50 ms bins during the following periods, precue (500 ms), postcue (800 ms), prereward (500 ms), and postreward (500 ms). Figure 5 displays the total percentage of M1 units that have significantly higher average FRs for rewarding (red) trials and nonrewarding (blue) trials (Wilcoxon signed-rank test, *p* < 0.05). In NHP A’s contralateral M1 (Fig. 5*A*, left column), 11.1% (39 of 352 for precue), 36.1% (127 of 352 for postcue), 26.7% (94 of 352 for prereward) of M1 units for the manual task had significantly higher FRs during rewarding trials than nonrewarding trials; whereas, 19.6% (69 of 352 for postreward) of M1 units had significantly higher FRs for nonrewarding trials than rewarding trials. For the observational task (Fig. 5*A*, right column), 48.5% for precue and 52.3% for postcue of M1 units had significantly higher FRs for rewarding trials than nonrewarding trials; whereas, 41.7% for prereward and 51.2% for postreward of M1 units had significantly higher FRs for nonrewarding trials as compared to rewarding trials. The observation task performed by NHP A was a fully predictable sequence of rewarding trials followed by nonrewarding trials, which explains the precue activity pattern (Tarigoppula et al., 2018). In NHP Z’s ipsilateral M1, during the manual task (Fig. 5*B*, left column), 19.2% for precue, 37.7% for postcue, and 33.6% for prereward had significantly higher spiking rates for rewarding trials than nonrewarding trials, but 31.5% for postreward period had higher FRs for nonrewarding trials than rewarding trials. Similar to the manual task, NHP Z showed a similar trend of population FRs as for the observational task. The detailed population FRs are shown in Figure 5*B*.

During grip force tasks, in NHP S’s contralateral M1 results (Fig. 5*C*), rewarding (red) trials as a whole always showed significantly higher FRs than nonrewarding (blue) trials for both the manual task (left column) and the observational task (right column). In NHP P’s contralateral M1 (Fig. 5*D*), during the manual task (left column), FRs for all periods were significantly higher for rewarding (red) trials than nonrewarding (blue) trials (Wilcoxon signed-rank, *p* < 0.05). For the observational task (right column) from NHP P’s contralateral M1 units, all periods except the precue period had higher FRs for rewarding trials than nonrewarding trials. The number of significant M1 units of NHP P’s neural spiking rate during observational tasks was lower compared with significance of manual tasks. Overall, the results show that neural rates are significantly higher in M1 when reward is expected.

### α Phase of LFP relates to neural spiking activity

**Figure 6.**
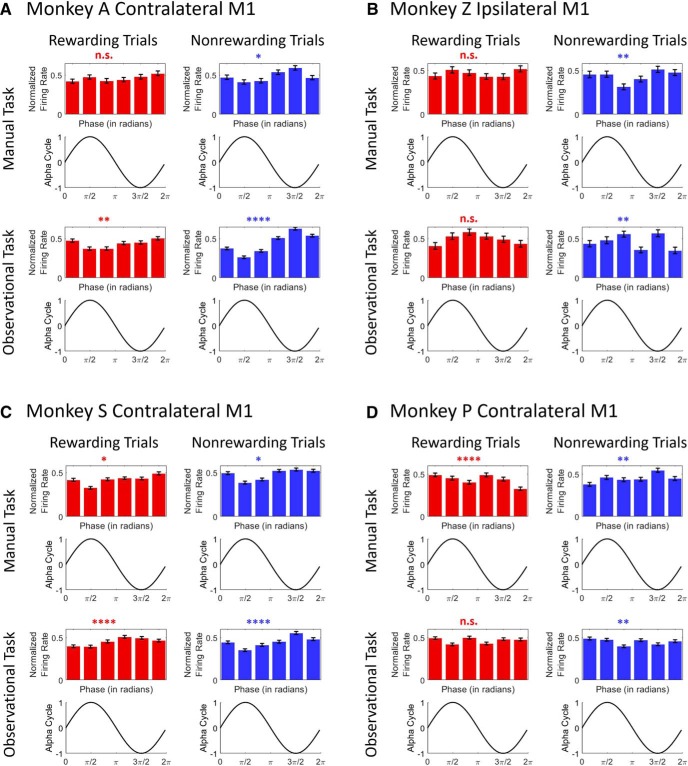
The relationship between neural firing and the α-band cycle during rewarding (left column in each subplot) and nonrewarding (right column in each subplot) trials across manual (upper row in each subplot) and observational task (lower row in each subplot) for contralateral (***A***, ***C***, ***D***) and ipsilateral (***B***) M1 cortices (****p < 0.0001, **p < 0.01, *p < 0.05; n.s., no significance; one-way ANOVA). Error bars in the bar plots represent SEM. ***A***, ***B*** are for the center-out reaching tasks, and ***C***, ***D*** are for the grip force tasks.

Previous work has shown a significant relationship between α power and neural FR such that FRs were highest at the trough and lowest at the peak of the α cycle (Haegens et al., 2011). Here we show some support for this observation, and demonstrate that it is strongest during the nonrewarding trials. Figure 6 shows the relationship between the α-band cycle and spike FR for rewarding (left column in each subplot) and nonrewarding (right column in each subplot) trials across manual (upper row in each subplot) and observational tasks (lower row in each subplot) for contralateral (Fig. 6*A*,*C*,*D*) and ipsilateral (Fig. 6*B*) M1 cortices. In the manual task (top row) for NHP A’s contralateral M1 (Fig. 6*A*), the FR is high around the trough (3π/2) of the α-band cycle, and low around the peak (π/2) of the cycle for nonrewarding trials (right column; *F*_(5,660)_ = 3.95, *p* < 0.05), whereas this was not observed for rewarding trials (left column; *F*_(5,744)_ = 1.36, *p* > 0.05). Similarly, for the observational task (Fig. 6*A*, bottom row), FR was high around the trough of the cycle, whereas it was low around the peak for nonrewarding trials (right column; *F*_(5,2040)_ = 25.79, *p* < 0.0001). We did not see the same pattern for rewarding trials (left column; *F*_(5,2040)_ = 3.7, *p* < 0.01). In NHP Z’s ipsilateral M1, during the manual task (Fig. 6*B*, top row), the FR was highest around the trough of the α cycle for nonrewarding trials (right column; *F*_(5,534)_ = 3.35, *p* < 0.01), but it was highest at the peak and lowest at the trough for rewarding trials (left column; *F*_(5,564)_ = 1.17, *p* > 0.05). During the observational task (Fig. 6*B*, bottom row), highest FRs were associated with the trough of the α cycle for nonrewarding trials (right column; *F*_(5,276)_ = 3.30, *p* < 0.01) but not for rewarding trials (left column; *F*_(5,294)_ = 1.63, *p* > 0.05).

We found similar results in the grip force tasks as seen in center-out reaching tasks. For the manual task (upper row), NHP S’s contralateral M1 (Fig. 6*C*) showed that the FR was high around the trough, and low around the peak of the cycle for nonrewarding trials (right column; *F*_(5,528)_ = 2.60, *p* < 0.05) whereas it was lowest around the peak of the α cycle for rewarding trials (left column; *F*_(5,678)_ = 2.43, *p* < 0.05). For the observational task (Fig. 6*C*, bottom row), FR was high at the trough of α cycle, and it was low around the peak for nonrewarding trials (right column; *F*_(5,1890)_ = 10.68, *p* < 0.0001). The FR was lowest at the peak for rewarding trials (left column; *F*_(5,2016)_ = 5.95, *p* < 0.0001). For NHP P’s contralateral M1 (Fig. 6*D*), manual task results (upper row) showed that FR was highest around the trough but not lowest around the peak for nonrewarding trials (right column; *F*_(5,1050)_ = 3.80, *p* < 0.01). The FR does not have the same trend for rewarding trials (left column; *F*_(5,1020)_ = 5.04, *p* < 0.001). For the observational task (bottom row), the highest and lowest FRs both for rewarding (*F*_(5,1350)_ = 1.72, *p* > 0.05) and nonrewarding trials (*F*_(5,1344)_ = 3.52, *p* < 0.01) were not associated with the peak and trough of the α cycle.

## Discussion

To determine the influence reward expectation has on M1, we recorded neural activity, single/multi-unit activity and LFPs from chronically implanted electrodes bilaterally in M1. We used multiple sensorimotor tasks where NHPs either made reaching or grasping movements, or observed such movements. We found several clear and reproducible patterns of activity between NHPs, cortical hemispheres and tasks. These patterns of activity included an increase in α power during cued nonrewarding trials, although the manual tasks still required targeted movements by the NHPs, and thus some level of attention. Nonrewarding trials also had stronger α-band SFC between spiking activity and the averaged LFP activity, and stronger α-phase γ-amplitude coupling as compared to cued rewarding trials.

The power of LFP oscillations is modulated by visual and auditory attention (Foxe et al., 1998; Thut et al., 2006; Rihs et al., 2009; Kerlin et al., 2010) and reward expectancy (van Wingerden et al., 2010; Lansink et al., 2016). Our PSD results showed a consistent reward-related decrease in the mean α power in bilateral M1 during postcue periods for rewarding as compared to nonrewarding trials for manual and observational tasks (Fig. 2). Evidence suggests that dopamine plays a critical role in the selection of targets for attention (Rose et al., 2010), and that injecting a dopamine D1-agonist into the prefrontal cortex of rats enhanced attentional accuracy, while a D1-antagonist led to decreased performance (Granon et al., 2000; Chudasama and Robbins, 2004). Furthermore, dopamine depletion has an influence on attention-deficit/hyperactivity disorder (ADHD; Jucaite et al., 2005; Silvetti et al., 2013). These studies suggest that changes in dopamine transmission or release could be responsible for changes in attention. Following this, it is expected that subjects pay more attention during rewarding trials to reach the targets (Dalley et al., 2002; Demiralp et al., 2007; Rose et al., 2010) compared to nonrewarding trials. Our findings are consistent with previous studies showing that dopamine depletion led to an increased power of LFP oscillations (Cassidy et al., 2002; Sharott et al., 2005; Costa et al., 2006; Kühn et al., 2008; Mallet et al., 2008; Lemaire et al., 2012). These results provide a clue toward explaining the relationship that exists between dopamine, reward expectation (or attention/motivation), and LFP oscillations in M1 cortex.

The phase of low-frequency oscillations modulate with the amplitude of high-frequency oscillations (Canolty et al., 2006; Tort et al., 2008; Canolty and Knight, 2010). Some evidence of phase-to-amplitudes comodulation has come from studies conducted across different frequency bands: δ-γ (Gross et al., 2013; López-Azcárate et al., 2013; Szczepanski et al., 2014), θ-γ (Bragin et al., 1995; Chrobak and Buzsáki, 1998; Canolty et al., 2006; Tort et al., 2008; Voytek et al., 2010; Lisman and Jensen, 2013; Voloh et al., 2015), α-γ (Osipova et al., 2008; Cohen et al., 2009; Voytek et al., 2010; Spaak et al., 2012; Yanagisawa et al., 2012; van Kerkoerle et al., 2014; Bonnefond and Jensen, 2015; Park et al., 2016; Seymour et al., 2017; Tzvi et al., 2018), and β-γ (de Hemptinne et al., 2013; Kim et al., 2015; Swann et al., 2015). In particular, some of these studies investigated α-γ comodulation in the visual cortices (Osipova et al., 2008; Voytek et al., 2010; Spaak et al., 2012; van Kerkoerle et al., 2014; Bonnefond and Jensen, 2015; Seymour et al., 2017), parietal-occipital areas (Tzvi et al., 2018), lingual gyrus (Park et al., 2016), and sensorimotor cortex (Yanagisawa et al., 2012). In our analysis, we found that reward expectation influenced comodulation between the phase of α-band (8–14 Hz) oscillations and the amplitude of γ-band (30–100 Hz) oscillations during the postcue period in M1 (Fig. 3). We found a higher strength of phase-to-amplitude comodulation in nonrewarding trials during both manual tasks and observational tasks while performing either a reaching (NHPs A and Z) or grasping task (NHPs S and P). In addition, increased α power led to stronger α-γ comodulation during all tasks; that is, the strength of α-γ comodulation was positively correlated with α power (Tort et al., 2008, 2013). These results are consistent with previous studies where stronger α-γ comodulation occurred while α-band activity increased (Osipova et al., 2008; Voytek et al., 2010; van Kerkoerle et al., 2014). Similar α-γ comodulation was modulated by different reward conditions in the nucleus accumbens of humans as well (Cohen et al., 2009).

As shown in Figure 4, a larger subpopulation of reward-modulated M1 units had significantly higher phase synchronization as measured with SFC between α oscillations and neural spikes in nonrewarding trials than in rewarding trials for all tasks. This indicates that higher phase-synchronization is seen in the presence of stronger α oscillations. Our SFC results are consistent with a previous study (Haegens et al., 2011), which showed that an increase of α power was associated with an increase in α-band SFC in premotor, motor, and somatosensory regions during a discrimination task. This suggests that stronger α oscillatory activity in M1 may give rise to a suppression of neural spiking and modulates the inhibitory timing of spiking (Klimesch et al., 2007; Jensen and Mazaheri, 2010; Mazaheri and Jensen, 2010; Jensen et al., 2012; Klimesch, 2012). Thus, a possible explanation for higher phase synchronization in the α-band between spikes and LFPs is that the lack of reward expectation leads to a decrease in dopamine associated motivation and/or attention, which amplifies α-band LFP oscillations, and as a result α oscillations lead to the inhibition and timing of neural spiking. It should be kept in mind that these results were seen when the NHPs had to manually perform the tasks, and thus they still needed to attend, and thus some of these differences are likely due to decreased motivational intensity and not only attention.

M1 FR was modulated by cued reward expectation as we have previously shown (Marsh et al., 2015). Similar reward-related modulation in observational tasks and manual tasks indicate that M1 units encode reward expectation (McNiel et al., 2016; Ramakrishnan et al., 2017; Tarigoppula et al., 2018). M1 units show mirror like modulation during the execution of reaching movements as well as observation of such actions (Tkach et al., 2008), and mirror neurons in the premotor cortex can be modulated by subjective value (Caggiano et al., 2012). Thus, there may be mirror like neurons modulated by value in M1 and S1 (Marsh et al., 2015; McNiel et al., 2016; Ramkumar et al., 2016; Ramakrishnan et al., 2017; An et al., 2018; Tarigoppula et al., 2018).

Figure 6 shows the possible existence of a relationship between α phase and neural FRs. During nonrewarding trials for both manual and observational tasks in contralateral M1 (NHP A for reaching tasks, and S for grasping tasks), FRs were high around the trough and low at the peak of the α cycle. These findings led to an initial conclusion that spiking was locked to the trough of α oscillations. These findings are consistent with a previous study (Haegens et al., 2011) where the FR was highest at the trough and lowest at the peak of the α cycle, and in general fit the pulsed-inhibition theory (Klimesch et al., 2007; Jensen and Mazaheri, 2010; Mazaheri and Jensen, 2010; Haegens et al., 2011; Jensen et al., 2012; Klimesch, 2012) which is a mechanism through which α oscillations suppress neural spiking activity. All of our results are in line with the idea that α oscillations serve an inhibitory function and that stronger α power reduces FRs (Haegens et al., 2011). However, unlike in Haegens et al., we saw slightly different α phase to FR results from our female NHPs in contralateral M1 units from NHPs P and AC8 (data not shown) as well as ipsilateral M1 units from NHP Z, where the FR was highest at the trough of the α cycle, but it was not lowest at the peak of the cycle. The reason for these results is not clear, but could be that ipsilateral M1 units were less significantly modulated by reward expectation than contralateral M1 units (Donchin et al., 1998; Cisek et al., 2003; Ganguly et al., 2009). Another interesting possibility is that this could be due to sex differences in dopamine receptor concentrations. Previous work has shown that female humans have much fewer dopamine receptors in the striatum as compared to males (Zaidi, 2010). These possibilities could have influenced the inhibition and timing of neural spiking with respect to the α cycle, although this is speculative.

The differences shown here between rewarding and nonrewarding trials have potential use toward the development of an autonomously updating BCI that utilizes the detectable reward modulation described in this paper and a BCI agent that updates autonomously via RL update rules. Toward this goal, our lab recently showed that integrated features of PSD and SFC yielded near-perfect classification accuracy (An et al., 2018) between rewarding and nonrewarding trials and could be used as a neural critic in autonomously updating BCIs (Bae et al., 2011; Sanchez et al., 2011; Tarigoppula et al., 2012, 2018; Marsh et al., 2015; An et al., 2018). Recently, we and others have shown that M1 directional tuning is also modulated by reward during manual (Ramakrishnan et al., 2017) and BCI control (Zhao et al., 2018), and that taking this into account could improve BCI control (Zhao et al., 2018). Further work is needed to incorporate our accurate classifier for an autonomously updating and accurate BCI system.

## Synthesis

Reviewing Editor: Trevor Drew, University of Montreal Decisions are customarily a result of the Reviewing Editor and the peer reviewers coming together and discussing their recommendations until a consensus is reached. When revisions are invited, a fact-based synthesis statement explaining their decision and outlining what is needed to prepare a revision will be listed below. The following reviewer(s) agreed to reveal their identity: Bruno Averbeck. Note: If this manuscript was transferred from JNeurosci and a decision was made to accept the manuscript without peer review, a brief statement to this effect will instead be what is listed below.

The authors have addressed my concerns. However, I think it would be worth including a portion of this explanation, quoted below, in the discussion, and incorporating some of this material into the intro, so readers will have a clear idea of the motivation for the paper.

“When we mention reinforcement learning (RL) in the first sentence of the abstract we are not talking about the NHPs brain, but rather of a brain-computer interface (BCI) agent that is made up of an RL agent, a line of work we have published on, including the 2015 Marsh paper reviewer #2 mentioned (An et al. 2018; McNiel et al. 2016; Marsh et al. 2015; Tarigoppula, Rotella, and Francis 2012; Bae et al. 2011). This RL agent can use reward expectation or state-value information from the user's brain, which is the environment to the RL agent in our case, in order for this BCI agent to maximize its reward, which again is a signal coming from the NHP users' brain. Thus, in this work, we were looking for reward modulation in the NHPs brain in order to move towards autonomously updating BCIs.”
